# Exploiting the Kumaraswamy distribution in a reinforcement learning context

**DOI:** 10.3389/frobt.2025.1589025

**Published:** 2025-10-30

**Authors:** Davide Picchi, Sigrid Brell-Çokcan

**Affiliations:** Chair of Individualized Production, RWTH Aachen University, Aachen, Germany

**Keywords:** machine learning, crane, construction, reinforcement learning, Kumaraswamy distribution

## Abstract

Mini cranes play a pivotal role in construction due to their versatility across numerous scenarios. Recent advancements in Reinforcement Learning (RL) have enabled agents to operate cranes in virtual environments for predetermined tasks, paving the way for future real-world deployment. Traditionally, most RL agents use a squashed Gaussian distribution to select actions. In this study, we investigate a mini-crane scenario that could potentially be fully automated by AI and explore replacing the Gaussian distribution with the Kumaraswamy distribution, a close relative of the Beta distribution, for action stochastic selection. Our results indicate that the Kumaraswamy distribution offers computational advantages while maintaining robust performance, making it an attractive alternative for RL applications in continuous control applications.

## 1 Introduction

The advent of artificial intelligence (AI) has had a profound impact on the approach to solving automation problems, leading to a radical paradigm shift in a wide range of fields. In the domain of automation and engineering, Reinforcement Learning plays an important role in developing new concepts in which an agent is capable of controlling dynamic systems without the necessity of solving or modeling them with differential equations or similar methods. In Reinforcement Learning, an agent can control a dynamic system by repeating actions and receiving a reward from the environment (Sutton and Andrew, 2018). By maximizing the reward, an agent can efficiently learn a specific task. This approach opens new avenues for automating tasks even in traditionally underdigitalized sectors like construction. Concurrent with this development, there has been a paradigm shift in various fields, including the construction sector, despite its relative underdigitalization. The capacity to train an agent capable of controlling a construction machine through the creation of a dynamic simulation environment and the iteration of agent actions over multiple time steps is now a possibility. The dynamic programming approach, developed many decades ago, has undergone extensive refinement and enhancement through the integration of numerous novel algorithms in recent years, particularly through the extensive integration of neural networks as universal function approximators (Sutton and Andrew, 2018). One of the most interesting and efficient algorithms used in many Reinforcement Learning applications today is the Proximal Policy Optimization (PPO) algorithm (Schulman et al., 2017). This algorithm is on-policy based, offering robust performance in many robotic tasks and rapid convergence. In a paper published by Hsu et al. (2020), the authors revisited the algorithm, introducing several modifications. One of these modifications was to use a Beta distribution instead of a Gaussian distribution for the stochastic agent. The authors demonstrated that the Beta distribution produced accurate results and, in many benchmark environments, these results were superior to those obtained by other algorithms (Hsu et al., 2020). The effort to improve performance results is exemplified in the research conducted by Chou et al. (2017) by incorporating the Beta distribution within gradient-based policies for continuous control in the context of deep Reinforcement Learning. The authors have documented higher scores and faster convergence properties compared to those achieved using a Gaussian distribution, across a range of different gradient policies (Chou et al., 2017). The objective of this study is to determine whether an agent has the capacity to acquire the skills necessary to operate a mini-crane and subsequently transfer that knowledge to a real crane. Simultaneously, an evaluation of the Kumaraswamy distribution as a potential replacement for the Gaussian or Beta distribution is warranted.

### 1.1 The Kumaraswamy distribution

In recent advances within the fields of Reinforcement Learning and control systems, the selection of probability distributions has emerged as a pivotal element in the effective modeling of action spaces. Conventionally, the utilization of squashed Gaussian distributions has been prevalent due to their capacity to model continuous variables with a defined support. However, this study proposes the use of the *Kumaraswamy* distribution as an alternative to the Beta distribution. The Kumaraswamy distribution is distinguished by its flexibility and computational efficiency and it is distinct from a Gaussian distribution in that it does not necessitate the application of non-linear functions to achieve bounded outputs, a process referred to as *squashing*. Instead, the Kumaraswamy distribution inherently operates within a defined interval, that is, (0,1) . This characteristic renders it particularly well-suited for tasks that require bounded actions. Furthermore, the Kumaraswamy distribution provides augmented parameterization alternatives through its two shape parameters, akin to the Beta distribution. These parameters enable a greater degree of control over skewness in comparison to a single variance parameter in Gaussian models. This flexibility enables more precise fitting to task-specific data distributions encountered in crane operations where varying environmental conditions may require adaptable response strategies. From a theoretical point of view, these advantages contribute to improved numerical stability and accelerated convergence rates during the training phases. The reduced complexity in sampling from Kumaraswamy facilitates efficient exploration strategies without incurring additional computational costs typical of squashing mechanisms applied to Gaussian samples.

Despite many similarities to the Beta distribution, such as two coefficients α,β or a , b for controlling their shape^1^, the Kumaraswamy distribution offers greater benefit, especially in combination with neural networks, for the following reasons:

1. It allows for a simplified form of log-probability computation, which is easier and faster to compute than the Beta distribution. This is a very important aspect, since the log-probability computation is fundamental in any gradient policy algorithm.
2. It has a closed-form derivative, which makes it easier and numerically stable for the gradient computation compared to the Beta distribution. This requirement is necessary for a differentiable policy parameterization.
3. Its entropy computation has a closed form solution, in contrast to the Beta distribution, which requires numerical integration. Entropy calculation is a prerequisite for most contemporary policy gradient algorithms.
4. It supports the reparameterization trick as shown in the work of Wasserman and Mateos (2024).
5. It is natively bounded in the range (0,1) . By constrast, the Gaussian is an unbounded distribution that needs to be *squashed* using the tanh function.
6. It offers greater robustness at the boundary (when: α,β<1 ), leading to more numerically stable behavior.

Mathematically, the PDF function of the Kumaraswamy distribution can be expressed by the following Equation 1:

$$f\left(x;\alpha ,\beta \right)=\alpha \beta x^{\alpha -1}{\left(1-x^{\alpha }\right)}^{\beta -1},\;0<x<1$$ (1)

While its CDF (Equation 2) and its inverse function (Equation 3) can be expressed as follows (Nalisnick and Smyth, 2016):

$$F\left(x;\alpha ,\beta \right)=1-{\left(1-x^{\alpha }\right)}^{\beta }$$ (2)

$$F^{-1}\left(u;\alpha ,\beta \right)={\left(1-{\left(1-u\right)}^{1/\beta }\right)}^{1/\alpha },\;u∼U\left(0,1\right)$$ (3)

Allowing for a computationally affordable reparametrization trick sampling from the Uniform distribution u∼U(0,1) :

$$x=F^{-1}\left(u;\alpha ,\beta \right)={\left[1-{\left(1-u\right)}^{1/\beta }\right]}^{1/\alpha }$$ (4)

A linear transformation (Equation 5) is needed to map the bounded action range (0,1) to the more common and symmetric action space (−1,1) :

a=2x−1 (5)

Leading to the following Jacobian (Equation 6) and logarithmic probability (Equation 7):

$$\left|\frac{dx}{da}\right|=\frac{1}{2}$$ (6)

$$\log\pi \left(a\right)=\log\alpha +\log\beta +\left(\alpha -1\right)\log a+\left(\beta -1\right)\log\left(1-a^{\alpha }\right)$$ (7)

### 1.2 The reparametrization trick in the Gaussian distribution

A considerable proportion of continuous control Reinforcement Learning algorithms yield action values that are distributed according to a Gaussian function. Subsequently, these values are processed through a tanh function (Equation 9) to align with the constraints of the action space (typically constrained to the interval (−1,1) ). Thus:

$$y∼N\left(\mu ,\sigma^{2}\right)$$ (8)

$$a_{t}=tanh\left(y\right)$$ (9)

In order to facilitate the flow of the gradient through stochastic nodes by sampling ϵ from a Uniform distribution (Equation 8) the sample y can be reparameterized as Equation 10:

$$y=\mu +\sigma \epsilon ,\;\epsilon ∼N\left(0,1\right)$$ (10)

where μ and σ are predicted by the policy network.

The inverse transformation of the squashed Gaussian is:

$$y=tanh^{-1}\left(a\right)=\frac{1}{2}\ln\left(\frac{1+a}{1-a}\right)$$ (11)

where *y* in Equation 11 is the reparametrized version of *y* in Equation 8, leading to the Jacobian (Equation 12) and the resulting log-probability formula (Equation 13):

$$\left|\frac{dy}{da}\right|=\frac{1}{1-a^{2}}$$ (12)

$$\log\pi \left(a\right)=-\log\sigma -\frac{1}{2}\log\left(2\pi \right)-\frac{1}{2}\left(\frac{a-\mu }{\sigma }\right)$$ (13)

As reported in Haarnoja et al. (2018).

### 1.3 The Beta distribution

For comparison, the PDF and the CDF of the Beta distribution are expressed as follows (respectively: Equations 14, 15):

$$f\left(x;\alpha ,\beta \right)=\frac{x^{\alpha -1}{\left(1-x\right)}^{\beta -1}}{B\left(\alpha ,\beta \right)}\;where\;B\left(\alpha ,\beta \right)=\frac{\Gamma \left(\alpha \right)\Gamma \left(\beta \right)}{\Gamma \left(\alpha +\beta \right)}$$ (14)

$$F\left(x,\alpha ,\beta \right)=\frac{B\left(x;\alpha ,\beta \right)}{B\left(\alpha ,\beta \right)}$$ (15)

While there is no simple closed form for the calculation of the inverse CDF, which complicates sampling via reparametrization tricks. The logarithmic probability formula can be expressed as follows.

$$\log\pi \left(a\right)=-\log B\left(\alpha ,\beta \right)+\left(\alpha -1\right)\,\log\left(\frac{a+1}{2}\right)+\left(\beta -1\right)\,\log\left(1-\left(\frac{a+1}{2}\right)\right)-\log2$$ (16)

where:

$$\log B\left(\alpha ,\beta \right)=\log\Gamma \left(\alpha \right)+\log\Gamma \left(\beta \right)-\log\Gamma \left(\alpha +\beta \right)$$ (17)

Which requires the computation of the log-gamma function logΓ(⋅) , leading to a computational overhead.

Equations 7, 13 illustrate a logarithmic probability calculation that incorporates logarithmic and exponential functions. Such computational burdens are less onerous than that of the log-probability computation of the Beta distributions (Equation 16), which implies the calculation of Equation 17. The Beta distribution incurs special-function evaluations (such as the log-gamma function), and its reparameterized sampling relies on two Gamma draws per action (Equations 16, 17), which are comparatively expensive. Conversely, the Kumaraswamy distribution avoids special functions entirely: its log-density and reparameterized sampler require only logarithms and power operations, with a closed form inverse CDF. Therefore, Kumaraswamy is typically considered computationally lighter than Beta, while remaining competitive with a squashed Gaussian, which is typically used in the context of Reinforcement Learning.

In the following Table 1 offers an overview about the different logarithmic probability computation formulas.

**TABLE 1:** Overview of the different logarithmic probability computation for three different distribution probabilities. The term a refers to the *action*.

Distribution	Logarithmic computational formula	Parameters determined by the policy
Gaussian	$\log\pi \left(a\right)=-\log\sigma -\frac{1}{2}\log\left(2\pi \right)-\frac{1}{2}\left(\frac{a-\mu }{\sigma }\right)$	μ,σ
Kumaraswamy	$\log\pi \left(a\right)=\log\alpha +\log\beta +\left(\alpha -1\right)\log a+\left(\beta -1\right)\log\left(1-a^{\alpha }\right)$	α,β
Beta	$\begin{array}{cc} & \log\pi \left(a\right)=-\log B\left(\alpha ,\beta \right)+\left(\alpha -1\right)\log\left(\frac{a+1}{2}\right)+\left(\beta -1\right)\log\left(1-\left(\frac{a+1}{2}\right)\right)-\log2\\ & \log B\left(\alpha ,\beta \right)=\log\Gamma \left(\alpha \right)+\log\Gamma \left(\beta \right)-\log\Gamma \left(\alpha +\beta \right) \end{array}$	α,β

To empirically validate the theoretical advantages discussed earlier, this work evaluates the efficacy of the Kumaraswamy distribution in two different environments. Models using Kumaraswamy, the squashed Gaussian and the Beta distributions are compared under identical conditions across various performance metrics. To ensure that observed differences are not due to hyperparameter tuning or random initialization, the evaluation protocol includes systematic tuning, retraining with different seeds, and swapping hyperparameters between distributions (only between the Kumaraswamy and the squashed Gaussian), thereby assessing raw performance, robustness, and generalization.

The protocol has been applied to two different environments:

1. A simulated mini-crane tasked with navigating from one position to another while circumventing obstacles.
2. The standard LunarLander environment provided by the Farama Foundation is a well-known common framework for testing Reinforcement Learning algorithms. The latter was utilized as a testbed for the proper implementation of the PPO algorithm.

The protocol evaluates along two axes:

1. Distribution: Gaussian vs. Kumaraswamy vs. Beta
2. Environment: LunarLander vs. mini-crane

and consists on the following steps:

1. Tuning of hyperparameters: For each pair (environment, distribution) the PPO hyperparameters have been optimized using the tree-structured parzen estimator (TPE) for 100 trials. During this phase, a fixed random seed was constantly used.
2. Retraining across seeds Subsequently, the best hyperparameters were used to retrain each configuration from scratch with 10 different random seeds.
3. Evaluation: Each trained model was evaluated in 25 independent episodes (with unseen seeds). The means and standard deviations across seeds were collected.
4. Swapping of hyperparameters: To test whether performance differences are due to distributional choice or hyperparameter bias, the Gaussian agents have been re-trained using the best Kumaraswamy hyperparameters and *vice versa*.
5. Repeat retraining and evaluation The swapped configurations were re-trained with 10 seeds and evaluated as described in step 2.

Because the LunarLander environment provides dense rewards using a different reward function than the mini-crane environment, the two cannot be compared directly on the same evaluation metric (in this case the average reward during the evaluation phase). For LunarLander only the reward-based metrics was collected, whereas for the mini-crane the success and collision rates. The following metrics were collected during the evaluation:

1. Success Rate (mini-crane only): Percentage of episodes in which the crane successfully reaches its objective without experiencing a collision. Calculated as the ratio of successful episodes to total evaluation episodes.
2. Collision Rate (mini-crane only): Percentage of evaluation episodes in which a collision occurs, defined as contact of any crane component with obstacles or the ground. Calculated as the ratio of episodes with collisions to total episodes.
3. Mean Evaluation Reward (LunarLander only): Average cumulative reward across all evaluation episodes.
4. Standard Deviation of Evaluation Rewards (LunarLander only): Variability of evaluation rewards across episodes, indicating stability performance of the learned policy.
5. Confidence Intervals for Mean Reward: For both environments, 95% confidence intervals were computed around the mean evaluation reward (or success rate) across seeds, using the standard error of the mean. This provides a statistical measure of reliability.
6. Area Under the Learning Curve (AUC): Computed over training timesteps and averaged across 10 seeds. The AUC aggregates learning efficiency, capturing not only the final performance but also the speed of convergence.
7. Completion time (mini-crane only): It represents the time requested by the agent to accomplish the task. The timesteps for every episode are limited to a maximum of 2048 steps.
8. Kullback-Leibler divergence: This metric represents the update of the policy gradient during the training phase.

The efficacy and numerical stability of the distributions are of critical importance, as they directly impact both the efficiency of training and the reliability of policy optimization. In contrast to conventional supervised learning scenarios, Reinforcement Learning algorithms necessitate the repeated computation of log-probabilities and entropy gradients during each update step. Consequently, distributions such as Kumaraswamy, which provide closed-form expressions, are particularly advantageous for stochastic policy parameterization in Reinforcement Learning agents. In addition, in continuous action spaces with bounded ranges, the Kumaraswamy distribution can directly model action probabilities without requiring costly transformations (e.g., tanh-squashing), which could enhance numerical stability and gradient estimation in policy-gradient methods. It is important to note that despite its interesting characteristics and properties, the Kumaraswamy distribution remains mostly under-used (Wasserman and Mateos, 2024).

### 1.4 The choice of the mini-crane scenario

The underlying rationale for the utilization of a mini-crane environment in this study is rooted in the potential for subsequent real knowledge transfer into practical applications and real mini-crane. A close examination of the current state of automation in the construction industry reveals that the integration of robotic technology into cranes is still in its nascent stages of development. This observation is based on extensive interactions with professionals in the construction sector, particularly those specializing in construction robotics. Therefore, the implementation of Reinforcement Learning in practical applications has the potential to enhance the automation level of construction machinery, thereby paving the way for future advancements in the construction sector. It is evident that Reinforcement Learning alone is insufficient to achieve and offer a comprehensive automation solution. In actual scenarios, the incorporation of anti-collision sensors and safety features is imperative if not mandatory. However, a trained Reinforcement Learning agent offers additional benefits and advantages to human operators, even if its role is initially limited to providing assistance without assuming complete control of the crane. Although full automation may require additional safety measures such as anti-collision sensors, which are beyond our current scope, the RL framework presented here lays foundational work toward intelligent assistance systems for crane operators.

### 1.5 Scope of this study

This paper addresses the problem of analyzing computational efficiency and robustness in continuous action-space Reinforcement Learning for construction robotics by evaluating the Kumaraswamy distribution as an alternative to commonly used Gaussian or Beta policies in the aforementioned mini-crane environment. The novelty of this work lies in:

1. Implementing a Kumaraswamy based stochastic policy within PPO for mini-crane control.
2. Providing a systematic comparison against Gaussian policies under identical experimental conditions.
3. Empirically analyzing performance across multiple metrics including implementation efficiency, stability, and computational costs.
4. Highlighting practical considerations for real-world deployment.

In the present work, the development of a stochastic agent based on the Kumaraswamy distribution embedded in a PPO algorithm has been undertaken, leading to a robust, high-efficiency stochastic policy capable of controlling a task where the dynamic system is represented by a mini-crane that picks up a load from one start point and moves it to a goal without hitting any obstacles and under joint limit constraints. This work expands the design space for continuous stochastic policies beyond Gaussian and Beta in robotics Reinforcement Learning, introducing a theoretically grounded yet computationally lighter alternative to the Beta distribution. Furthermore, it introduces an underexplored, yet theoretically promising, distribution to Reinforcement Learning control tasks where bounded actions are required.

## 2 Literature review

The use of Reinforcement Learning (RL) in construction engineering is a subject that has been explored to some extent. Previous studies have investigated the application of Reinforcement Learning in training an agent to operate a crane in a virtual environment. This paper (Cho and Han, 2022) proposes the use of Reinforcement Learning to reduce and optimize lifting times using a tower crane in a virtual 3D environment. The tower crane is trained to perform autonomous and continuous actions by controlling the velocities of rotation, lifting, sliding, and other related processes. The authors demonstrated the efficacy of the agent in generating trajectories that optimize lift plans and crane operations, thereby avoiding collisions with obstacles. In their work, they benchmarked two different algorithms: an on-policy (PPO) and an off-policy (SAC) algorithm, by using different reward function combinations and tasks. The results showed that while both PPO and SAC agents were effective across different tasks, PPO achieved higher performance metrics than SAC in several scenarios (Cho and Han, 2022).

Another intriguing piece of research was presented by Keita et al. (2020), who used Reinforcement Learning to automate the movements of a crane and an excavator by behavior cloning. In their environment, they trained an agent for two distinct tasks: the primary task was to minimize the oscillation of a load, while the secondary task was to maximize the excavation of soil in a single operation. The study demonstrated the applicability of Reinforcement Learning in construction sites and the ability to train agents for specific tasks. A more sophisticated concept was presented in another study, in which Reinforcement Learning control was applied to a forestry crane manipulator (Andersson et al., 2021). The log grasping motion, combined with an energy optimization goal, constituted a significantly more complex task. In fact, Andersson et al. had to train their agent for approximately 20 million steps using curriculum learning. Thus, it can be concluded that the agent began with straightforward tasks and progressed to more intricate activities as its success rate increased over experience. The results obtained claim a success rate of 97% when using an on-policy algorithm, such as PPO, to train the agent. The mentioned work is unquestionably one of the most significant papers that provided the inspiration for our work.

It should also be noted that the authors were able to incentivize energy optimization by embedding it in the reward function (Andersson et al., 2021). Another paper examines the application of Reinforcement Learning in construction environments characterized by obstacles (Xiao et al., 2023). In that work, the authors Xiao et al. trained a PPO agent for controlling a 3D lift path planning of a crane during unloading and loading operations. The authors considered two different cases, which are recurrent in the aftermath of an earthquake scenario: one case without and one case with obstacles between the initial and final position, that could hinder the agent in its performance sensibly. The paper (Xiao et al., 2023) demonstrates that appropriate training leads to an agent policy that can achieve scheduled goals and reduce swing load oscillations within time constraints.

In their work, Kai et al. (2022) proposed an approach to crane scheduling operation model using deep Reinforcement Learning, implementing a Q-learning algorithm with deep neural networks. The authors treated a steel fabrication process, where two cranes need to perform a sequence of actions along the process, as a scenario. Notably, the authors did not employ more sophisticated algorithms such as TD3, PPO, SAC, or A2C. Nevertheless, they successfully trained an agent that was capable of achieving the objectives in 11.52% less time and reducing the collision time of crane routes by almost 57% (Kai et al., 2022). This resulted in enhanced efficient scheduling management performed by an AI agent.

The crane scheduling process is the primary focus of this other paper, in which the authors implemented a dynamic environment that has significantly enhanced the efficiency of automated storage yards through the use of twin automated stacking cranes (Xin et al., 2023). In this extensive and highly complex article, the authors demonstrate that an agent can learn sophisticated scheduling policies and concurrently generalize its problem-solving capabilities, thereby enabling deployment in unseen scenarios of various scales or distributions (Xin et al., 2023). A notable aspect of this work is the utilisation of the *masked self-attention* mechanism for training the agent within the framework, which has been shown to yield high-quality policies for the given task. The self-attention mechanism forms the foundation of the *transformer* architecture (Vaswani et al., 2023), a significant advancement in the field of AI, leading to the development of tools such as GPTs, image and music creation programs, and numerous other applications.

The following Table 2 offers a summary of the lacunae and the objectives pursued by the aforementioned studies.

**TABLE 2:** Overview of the field of publications on the topic Reinforcement Learning in construction. The following abbreviations are used: PPO, Proximal Policy Optimization; SAC, Soft Actor Critic; BC, Behavior Cloning; FS, Frame Skipping.

Study	Application	RL algorithm	Stochastic distribution	Eval. Metrics	Main contribution
Cho and Han (2022)	Tower Crane	PPO/SAC	Gaussian (assumed)	z-score standarization across different metrics	Realistic lifting time estimation
Keita et al. (2020)	Crane and excavator	PPO, BC, FS	Gaussian (assumed)	Success Rate	Shows policy impact varies by machine type
Andersson et al. (2021)	Forestry Crane Manipulator	PPO	Gaussian	Success Rate	Explores energy policy effects
Xiao et al. (2023)	Robotic Crane	PPO	Gaussian	Success Rate (with/without obstacles)	Tests obstacle handling in crane control
Kai et al. (2022)	Multi-crane scheduling	Deep RL	Gaussian (assumed)	Completion time, travel distance	Efficient multi-crane scheduling framework
Xin et al. (2023)	Automated Stacking Cranes	PPO	Gaussian (assumed)	Wait time, run time minimization	RL method for Automated Stacking Cranes scheduling

The majority of previous works rely on Gaussian policies because of their mathematical convenience. However, these policies are deficient in their inability to address the limitations imposed by bounded action spaces. A limited number of studies have examined alternative double-bounded distributions, such as the Beta distribution (Hsu et al., 2020) in combination with a PPO policy algorithm. This alternative to a Gaussian distribution is of interest. However, it should be noted that the integration of PPO with a Beta distribution does incur a computational overhead that arises from log-probability calculations intrinsic to Beta distributions, as reported in Table 1. Moreover, the aforementioned study (Hsu et al., 2020) has not concentrated on construction robotics contexts, but rather has examined the benefits of the Beta distribution in a Reinforcement Learning context. The Kumaraswamy distribution constitutes an alternative option, exhibiting a similarity to the Beta distribution while avoiding the computational overhead characteristic of the latter. The present study proposes a methodology to address the aforementioned gaps using a systematic benchmarking process. This process involves the Kumaraswamy distribution being compared against established alternatives within a realistic mini-crane simulation environment.

## 3 The environment

**FIGURE 1:**
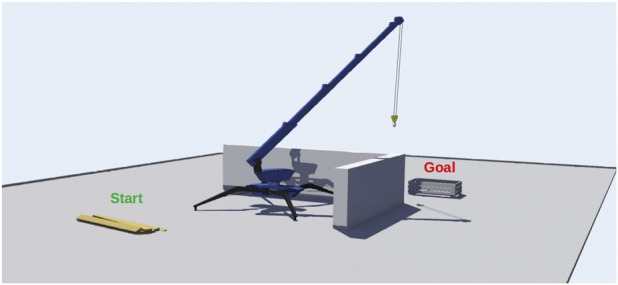
Simulation of a mini-crane going from one starting to a goal position.

The present study investigates and analyzes the applicability of the Kumaraswamy distribution applied to a mini-crane, which is tasked with a fundamental yet elementary undertaking: navigating its hook from a designated starting point to a predetermined goal position, while circumventing obstacles and avoiding collisions with the ground (Figure 1). This elementary task has been replicated within the simulation program Webots^2^, an open source software mostly used in robotic applications that allows virtual experimentation in dynamic environments, including collisions between objects.

**FIGURE 2:**
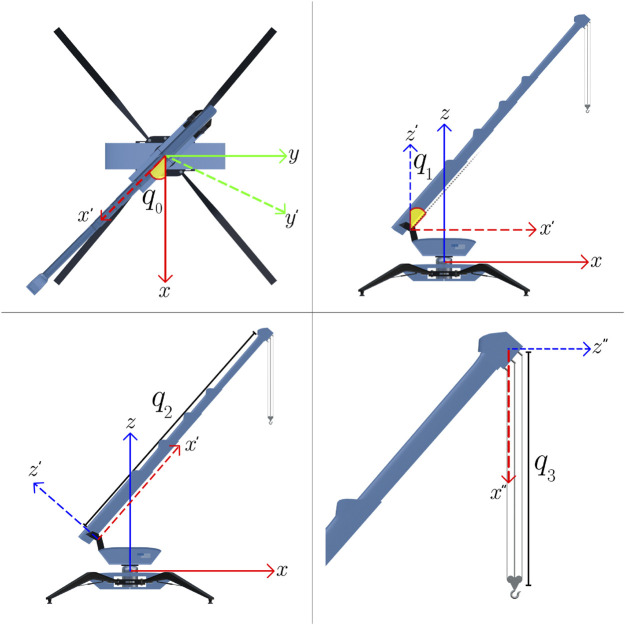
Joint positions of the mini-crane.

The crane possesses a total of four degrees of freedom around four joints: while the body is fixed to the ground, the upper part of the body can rotate $\left(q_{0}\right)$ around the azimuth axis (Figure 2). Furthermore, the boom can be adjusted to change its angle $\left(q_{1}\right)$ and extend as a telescopic arm $\left(q_{2}\right)$ . Finally, the hook movement consists of a linear translation $\left(q_{3}\right)$ perpendicular to the ground within predefined hard limits (not above the boom tip and not below the ground). Thus, the coordinates of the described system are defined by virtual sensors and, in a real-case scenario, can be provided over an interface connected to sensors on the real machine. The joint coordinates define at the same time the observed vector that corresponds to the state $\left(s_{t}=\left[q_{0},q_{1},q_{2},q_{3}\right]\right)$ of the system, as depicted in Figure 2.

The choice of spherical coordinates is based on the physical configuration of standard mini-cranes, which includes the rotational base joint, the angle of the boom, the telescopic extension, and the length of the rope. Direct modeling of these actuators’ native domains has been shown to simplify the handling of kinematic constraints in comparison to the mapping of Cartesian outputs back into joint space. This is due to the fact that mapping Cartesian outputs back into joint space can result in highly nonlinear and non-invertible behavior in the vicinity of the workspace limits. Therefore, the definition of both state/action spaces in native joint coordinates is consistent with hardware reality, such as real sensors..

**FIGURE 3:**
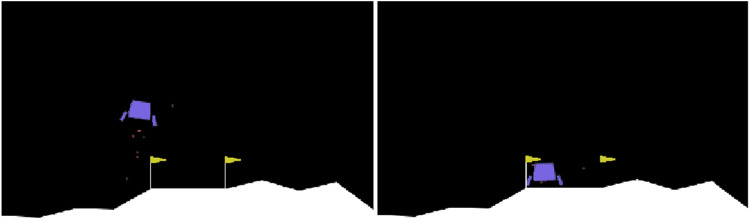
The LunarLander environment.

In the present study, the agent is trained in the following scenarios:

1. In the context of the LunarLander environment, the agent is tasked with the objective of landing a rocket-propelled lander by exercising control over two engines: a lateral engine and a main engine (Figure 3). The observation space comprises the two-dimensional coordinates of the ship, its linear and angular velocities, and the status of the legs’ contact with the ground. The action space is continuous. The reward function structure of the aforementioned environment consists of the distance to and from the landing pad, the landing speed, the tilt angle of the ship, the engine status, and the contact leg/ground. An additional positive/negative reward is granted for landing safely or crashing the ship on the landing pad.
2. In the context of the miniature crane environment, the simulation of a crane is required to execute the aforementioned task. The observation vector contains the joint coordinates and the absolute coordinates of the target, thus: $s_{t}=\left[q_{0},q_{1},q_{2},q_{3},x_{g},y_{g},z_{g}\right]$ . This case is likely to be the easiest to implement in a future scenario involving a real mini-crane, as the joint positions are simply the sensor values that can be gathered directly from a hardware interface, given that hardware sensors detect the joint state. Due to the observation vector not containing all the information of the system state, such as the joint velocities, this simple scenario corresponds basically to a POMDP (Partially Observable Markov Decision Processes).

In both cases described above, neural networks are trained solely based on the reward function r from their respective environments; the transition matrix p is not modeled, as this work follows a model-free approach. In the mini-crane environment, the observation vector is normalized and constrained to the range [−1,1] to prevent numerical instability or imbalances in network weights. No such normalization is applied to observations in the LunarLander environment.

For the mini-crane environment, four distinct actions define the action-space A : $a_{0}$ for the crane rotation, $a_{1}$ for the boom inclination, $a_{2}$ for the boom extension, and $a_{3}$ for the hook rope, and all are normalized and constrained in the range [−1,1] as for the state-space. Additionally, state variables are bounded to avoid self-collisions or physically impossible joint configurations, reflecting real-world constraints. For example, the coordinate $q_{0}$ is limited to the range $\left[-{q_{0}}_{\min},{q_{0}}_{\max}\right]$ , the inclination $q_{1}$ is limited to $\left[{q_{1}}_{\min},{q_{1}}_{\max}\right]$ , the extension $q_{2}$ is limited to $\left[{q_{2}}_{\min},{q_{2}}_{\max}\right]$ , while the hook height $q_{3}$ must remain between the tip of the boom and the ground.

**FIGURE 4:**
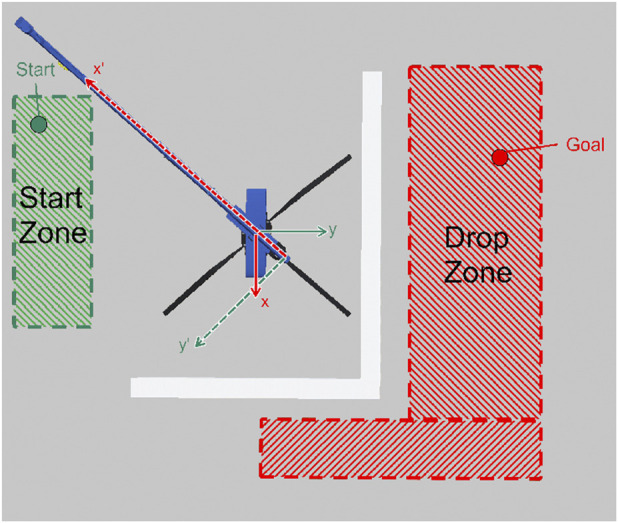
Overview of the zones, where the start and goal position is sampled from at the beginning of every episode.

In order to ensure that the problem is generalized and the robustness of the agent is improved, it is essential to initiate each episode by randomly determining the starting and goal positions in the fixed reference frame. This randomization has been achieved using a uniform distribution, which means that every possible position within the defined space has an equal likelihood of being selected. In doing so, the agent is exposed to a wide variety of initial conditions and target destinations, which helps to develop a more adaptable and resilient policy. The process is illustrated in Figure 4 which shows the areas of generation of start and target positions at the beginning of each episode.

For an episode to be designated as having a positive conclusion and to end within the time horizon, it is necessary for the mini-crane to move its load to a position close to the drop point (Figure 4). The episode ends only successfully when the Euclidean distance between the drop point and the load is ≤0.1 . In order to achieve this condition, the crane is forced to maneuver the hook below the height of the wall.

### 3.1 On-policy algorithm

Within the domain of Reinforcement Learning, any environment can be conceptualized as a Markov Decision Process (MDP). An environment fulfills the Markov property when the current state fully captures all relevant past information needed to take the next action. Furthermore, an MDP is characterized by the collection of a *trajectory* within a specific time horizon T comprising the following elements ( S , A , p , r , γ , ρ ), where: S represents a set of possible states, A represents a set of stochastic actions $a∼\pi_{\theta }\left(s\right)$ , $p=p\left(s_{t+1}|s,a\right)$ is the transition probability distribution of the system, r is the reward function, γ is a scalar value representing the discounting factor (S and G, 2018). In the course of an episode, the *agent* has the ability to collect the actual state $s_{t}$ at any given time step t (where the system is initially in the state $s_{0}$ ). The action $a_{t}$ to be taken according to a stochastic policy $\pi_{\theta }$ at any time t , resulting in a new state of the system $s_{t+1}∼p\left(s_{t+1}|a_{t}\right)$ and a new reward $r=r\left(s_{t},a_{t}\right)$ based on the state and action taken according to the policy $\pi_{\theta }$ . In the field of Reinforcement Learning, a prevalent approach involves the development of algorithms capable of generalizing across a range of environments. These algorithms are designed to consider a distribution of environments that encompasses diverse settings and configurations. The overarching objective of Reinforcement Learning is to determine the optimal policy $\pi_{\theta }^{*}$ that maximizes the expected reward of an episode, as shown in the following (Equation 18).

$$\underset{\theta }{\text{maximize}}\;E_{\pi_{\theta }}\left[\overset{T}{\underset{t=0}{\sum }}\gamma_{t}r_{t}\right]$$ (18)

The agent is designed to learn to generalize and determine actions in a variety of situations, thereby improving its resilience to potential obstacles and other challenges. In this research, the primary focus is on the PPO algorithm, used to train a policy that determines the subsequent action to be executed following a training phase extending over 1.2 million timesteps within the environment. The total loss $L_{t}^{POLICY+VF+H}$ is constituted by the cumulative effect of loss functions, which surrogates the policy, the value, and the entropy functions, as delineated in the Equation 19 and explained in more detail in the original work of Schulman et al. (2017):

$$L_{t}^{POLICY+VF+H}\left(\theta \right)={\hat{E}}_{t}\left[L_{t}^{\mathrm{POLICY}}\left(\theta \right)-c_{1}L_{t}^{VF}\left(\theta \right)+c_{2}H\left(s_{t};\theta \right)\right]$$ (19)

where: $c_{1},c_{2}$ are positive coefficients treated as hyperparameters while $H\left(s_{t};\theta \right)$ represents the entropy of the Kumaraswamy distribution and is given the following (Equation 20):

$$H\left(x;\theta \right)=-\int_{0}^{1}\alpha \beta x^{\alpha -1}\,{\left(1-x^{\alpha }\right)}^{\beta -1}\,\left[\log\alpha +\log\beta +\left(\alpha -1\right)\,\log x+\left(\beta -1\right)\,\log\left(1-x^{\alpha }\right)\right]\,dx$$ (20)

where: α,β are the coefficients for controlling the Kumaraswamy distribution. As there is no simple closed-form solution to the above equation, there are many estimates of entropy for the Kumaraswamy distribution, which have been evaluated by Al-Babtain et al. in their work (Al-Babtain et al., 2021). One of the most important issues dealing with such equations is the numerical instability due to integral computation. For this reason, the approximation used in this work is expressed in the following form, (Equation 21), which represents the differential entropy for that distribution and was found to be numerically stable:

$$H\left(•;\theta \right)=1-\beta +\left(1-\alpha \right)\left(\psi^{\left(0\right)}\left(\beta^{-1}+1\right)+\gamma_{em}\right)-\log\alpha -\log\beta$$ (21)

where: $\psi^{\left(0\right)}$ represents the digamma function, while $\gamma_{em}$ represents the Euler-Mascheroni constant (Wasserman and Mateos, 2024). Calculating the gradient in a gradient-based policy algorithm requires the calculation of logarithmic probability. For the Kumaraswamy distribution, the log-probability is given by the Equation 7 and represents a closed-form solution that does not require integration, although numerical instability may occur as the argument of the logarithm approaches 0. In order to compensate for the above issue, the implementation proposed by Wassermann et al. has been adopted (Wasserman and Mateos, 2024).

**FIGURE 5:**
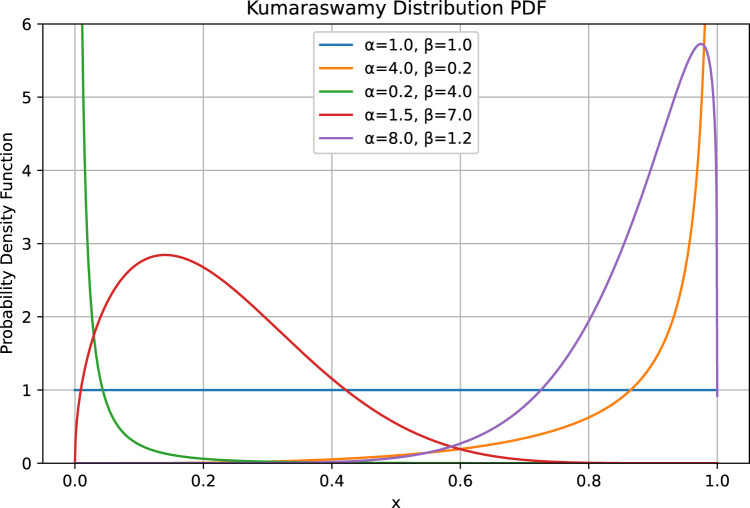
Behaviour of a Kumaraswamy distribution with different coefficients.

The parameters (α,β) are determined by the stochastic policy neural network that represents the agent. As previously stated, the Kumaraswamy distribution bears a strong resemblance to the Beta distribution. A comparison of the two distributions reveals that they are characterized by similar distributional properties and are both suitable for continuous probability control problems. Furthermore, both are controlled by two coefficients that determine the shape of the distribution, as depicted in the following (Figure 5).

In the event that either parameter α<1 or β<1 , the distribution becomes *peaked* resulting in the agent’s loss of stochasticity and the emergence of deterministic behavior. Conversely, when both α=β=1 , the distribution degenerates into a Uniform one. For this reason, great attention is paid to the output of the agent’s neural network so that the constraint α≥1 and β≥1 is strictly satisfied by using the *softplus* function as the activation layer and adding 1 to its output. In typical circumstances, the coefficients α and β are determined by a learned policy, resulting in a distribution that exhibits “skewness” in the direction of the sampled action. Once sampled, a remapping of the action (0,1)→[−1,1] through a linear function is necessary to match the action space modeled by both environments.

### 3.2 Reward and coordinate reference system

As stated previously, the reward depends on the state of the system at any given time step and on the specific policy. In this research, experiments with numerous dense reward functions for the mini-crane environment were conducted, meticulously ensuring that each function was differentiable and continuous in its domain. Defining d as the Euclidean distance between the hook and the goal position in space, the optimal performance was achieved through the implementation of the following reward function (Equation 22):

$$r=r_{e}+r_{b}+r_{p}$$ (22)

where: $c_{3}$ is a positive constant, while d is the absolute Euclidean distance between the hook and the goal, thus: $\left(d_{t-1}-d_{t}\right)$ is positive when the hook is moving toward the goal position, negative otherwise. Furthermore, the reward function is augmented by a positive bonus whenever the hook reaches the goal position within the aforementioned tolerance. In contrast, a negative bonus is appended to the reward whenever the hook or boom collides with a wall, reaches the joint limits, or makes contact with the ground. Experiments in which a time penalty was incorporated into the reward function did not yield superior results; thus, the time factor was neglected in the reward calculation.

In general, the calculation of the Euclidean distance between two points in space is possible even when their position is expressed in spherical coordinates, as depicted in general (Equation 23), under the assumption that two points $p_{1},p_{2}$ have the same reference frame:

$$\|p_{1}-p_{2}\|=\sqrt{\rho_{1}^{2}+\rho_{2}^{2}-2\rho_{1}\rho_{2}\left(\cos\theta_{1}\cos\theta_{2}\cos\left(\phi_{1}-\phi_{2}\right)+\sin\theta_{1}\sin\theta_{2}\right)}$$ (23)

**FIGURE 6:**
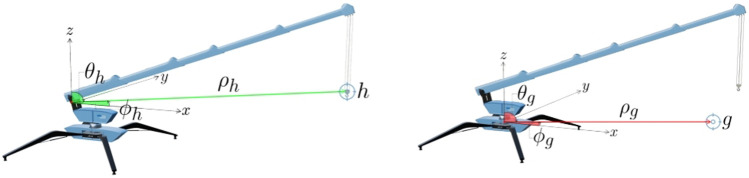
It is imperative to transform the hook coordinate system into the goal one, as the hook reference system is rotating with respect to the goal one.

In the context of the mini-crane studied in this work, a nuanced problem arises related to the coordinate system that is associated with the crane’s body. Specifically, this coordinate system undergoes rotational motion relative to a fixed reference coordinate system, which is used to define the positions of the goal and the surrounding walls. This means that as the crane operates, its coordinate system rotates, introducing complexity in how positions and movements are represented and calculated with respect to the stationary reference points of the goal and walls, as depicted in Figure 6.

This misalignment can introduce errors into distance computations and thus into reward assignment, unless all positions are transformed into a common reference frame before calculating Euclidean distances and lately the reward. Consequently, it is necessary to transform the hook position into the reference frame of the goal position, which is assumed to be fixed with the world. Then, the Euclidean distance between the two points can be calculated by using the Euclidean norm. The formula for distance computation is as follows:

$$d=|p_{1}-p_{2}\|=\sqrt{{\left(p_{1_{x}}-p_{2_{x}}\right)}^{2}+{\left(p_{1_{y}}-p_{2_{y}}\right)}^{2}+{\left(p_{1_{z}}-p_{2_{z}}\right)}^{2}}$$ (24)

## 4 Training the agent

In the present work, the PPO algorithm (Schulman et al., 2017) is implemented with three different stochastic agents: one based on a Gaussian distribution, one on a Kumaraswamy and the last on the Beta distribution. This process was performed separately for both the LunarLander environment and the mini-crane environment. The parameters of each distribution ( μ,σ for the Gaussian and α,β for the Kumaraswamy and for the Beta) were determined by two distinct neural networks. For the actor network, a shared base layer but separate output heads were used to predict either (μ,σ) or (α,β) , depending on the chosen distribution. All implemented neural networks use tanh as activation function between dense layers and employ *Adam* as the optimizer.

As stated previously, the input to neural networks is the observation vector $s_{t}$ , which comprises the crane joint values and the 3D coordinates of the target for the mini-crane environment. These data are concatenated into a single vector and normalized to the range [−1,1] before being passed to the policy network for action selection. For the LunarLander environment, the observation vector is passed unchanged from the environment to the neural networks. No normalization is applied, as all the distributions investigated in this work showed a very high degree of robustness, making a normalization superfluous.

**FIGURE 7:**
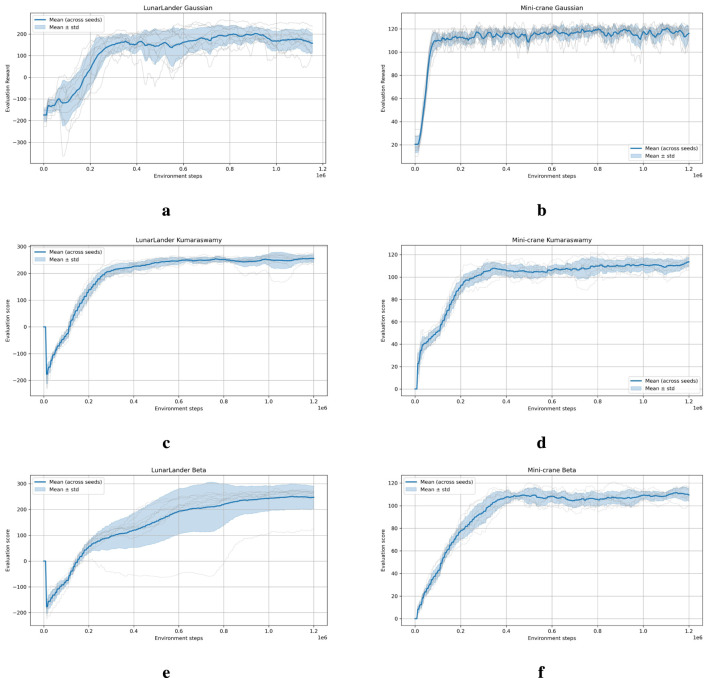
Training learning curves for the six considered scenarios. Each training was performed keeping the best hyperparameters and re-training the model with 10 different seeds. **(a)** LunarLander environment with Gaussian distribution. **(b)** Mini-crane environment with Gaussian distribution. **(c)** LunarLander environment with Kumaraswamy distribution. **(d)** Mini-crane environment withKumaraswamy distribution. **(e)** LunarLander environment with Beta distribution. **(f)** Mini-crane environment with Beta distribution.

The training phase began with the identification of optimal hyperparameters for a given distribution and environment, as reported in Table 3. Subsequently, the agent has undergone retraining with ten distinct seeds while maintaining the same hyperparameters. The learning curves resulting from the best set of hyperparameters can be found in the following Figure 7 for performance comparison. The mini-crane and LunarLander environments possess different reward functions. Therefore, a direct comparison between the range of rewards on the y -axis of one environment and the other is not possible.

**TABLE 3:** Overview of the performed training phase. Every environment has been trained once with one distribution. Once the best hyperparameters have been found, the same environment has been re-trained using the other distribution but keeping the same optimal hyperparameters (with the only exception of the Beta distribution). The swap test is for ensuring robustness.

Environment	Distribution	Tuning	Purpose	Swapped distribution
LunarLander	sq. Gaussian	TPE	Identification best hyperparameters	Kumaraswamy
LunarLander	Kumaraswamy	TPE	Identification best hyperparameters	sq. Gaussian
LunarLander	Beta	TPE	Identification best hyperparameters	—
Mini-crane	sq. Gaussian	TPE	Identification best hyperparameters	Kumaraswamy
Mini-crane	Kumaraswamy	TPE	Identification best hyperparameters	sq. Gaussian
Mini-crane	Beta	TPE	Identification best hyperparameters	—

The configuration of the reward function constitutes a pivotal element within the Reinforcement Learning framework, exhibiting considerable variability across different environments. In the present study, a series of dense reward functions were examined. It was determined that employing the raw Euclidean distance directly or as a negative exponent of any positive base presented substantial challenges to the agent in learning an acceptable policy. Conversely, the absolute Euclidean distance between the hook and goal position lacks sufficient signal strength to facilitate the agent’s learning of an appropriate policy. Consequently, the disparity in Euclidean distance between one timestep and the subsequent one is multiplied by a positive factor $c_{3}$ , which provides a reliable learning signal for the agent. Incorporating a negative time-based penalty to enhance the learning process was introduced many times during the implementation. However, the temporal dimension necessitates appropriate scaling and introduces a new parameter into the reward function, without showing any benefit to the learning signal strength. Consequently, the time penalty was eliminated in the final reward function, which has been reported in Table 4.

**TABLE 4:** Overview of the reward function structure. The values: $c_{3},c_{4},c_{5}$ represent constants.

Reward	Description	Value
$r_{e}$	Variation of the Euclidean distance between hook and goal from the previous to the actual timestep	$c_{3}\;\left(d_{t-1}-d_{t}\right),\;c_{3}>0$
$r_{b}$	Bonus when goal is reached	$\left\{\begin{array}{cc} c_{4}>0,\; & \text{if goal reached}\\ 0,\; & \text{otherwise} \end{array}\right.$
$r_{p}$	Penalty when collision occurs	$\left\{\begin{array}{cc} c_{5}<0,\; & \text{if collision occurs}\\ 0,\; & \text{otherwise} \end{array}\right.$

For agents based on Kumaraswamy and Beta distributions, a *Softplus* activation function is applied after the final dense layers of the actor network. Furthermore, a constant value of 1 is added to ensure that the coefficients α and β of both distributions do not approach extreme values, which could otherwise result in numerical instability during computation, as shown in Figure 5. For the optimization phase, the TPE algorithm has been utilized to determine the following hyperparameters: the actor learning rate, the critic learning rate, the scale factor for the value function $c_{1}$ , $\lambda_{\mathrm{gae}}$ , the distribution entropy coefficient $c_{2}$ , the discount factor γ , the batch size, and the network architecture for the actor and critic.

The reward function and the corresponding environments were kept constant throughout all phases of training, evaluation, and testing.

The experimental results presented in this study are the product of a training phase of 1.2 million timesteps.

## 5 Results

As illustrated in Table 5 the performance metrics for the LunarLander environment are compared for agents that were trained with a squashed Gaussian, Kumaraswamy and Beta as a policy distribution, validating the functionality of the implemented algorithm. For each distribution, the optimal top-3 hyperparameters were identified through the employment of TPE followed by a performance evaluation across 10 distinct random seeds over the course of 25 episodes. To assess robustness, the optimal hyperparameters were then applied to the alternate distribution (swap test).

**TABLE 5:** Mean and standard deviation of the results obtained by testing the LunarLander environment with 10 different random seeds over 25 episodes, after identifying the best hyperparameters. The reported metrics in the table represent the average score during the evaluation process across the 10 seeds. Subsequently, the base distribution was replaced and the model retrained using the same hyperparameters. The table shows, on the left, the new results after swapping the distributions.

Dist	Rank	Test score μ±σ	AUC μ±σ	Swap dist	New test score μ±σ
sq. Gauss	#1	244.88±10.929	182.31±11.38	Kumaras	242.60±18.96
sq. Gauss	#2	236.78±12.06	n.a	Kumaras	227.71±13.29
sq. Gauss	#3	236.71±12.06	n.a	Kumaras	236.34±13.39
Kumaras	#1	240.82±23.76	192.97±23.04	Sq. Gauss	245.49±18.44
Kumaras	#2	228.81±19.37	195.60±8.60	Sq. Gauss	237.99±17.03
Kumaras	#3	233.86±21.42	192.50±16.04	Sq. Gauss	247.42±15.18
Beta	#1	219.79±5.22	146.51±51.05	No swap	—
Beta	#2	233.01±14.51	118.67±92.10	No swap	—
Beta	#3	220.96±14.50	171.56±14.2	No swap	—

As reported in the Table 5, both distributions yield comparable mean test scores (μ) with relatively low standard deviations (σ) , suggesting consistent performance in varying random seeds. The top-ranked squashed Gaussian agent attains a score analogous to its Kumaraswamy and Beta counterpart. The Area Under Curve (AUC) values, consisting of the average evaluation score over training steps, demonstrate comparable trends between the distributions, with a slightly lower mean values obtained by the Beta distribution.

In the context of the LunarLander environment, the process of swapping distribution and re-training the agent with an alternative distribution while maintaining the previously optimized hyperparameters does not result in a substantial degradation of the agent’s performance. A notable observation is the improvement in the mean score when transitioning from Kumaraswamy to squashed Gaussian. The result indicates that, under optimal conditions, both distributions demonstrate comparable levels of expressiveness for the designated task. Furthermore, the optimized hyperparameters exhibit a satisfactory degree of generalization across both distributions.

Table 6 summarizes analogous experiments conducted in the mini-crane environment, reporting success rates (target reached), collision rates, and AUCs. Agents employing the squared Gaussian distribution consistently outperform those based on a Kumaraswamy and the Beta distribution in terms of target-reaching success rate while maintaining minimal collision rates. The AUC metric further supports this trend: squashed Gaussian policies achieve higher values relative to their counterparts.

**TABLE 6:** Mean and standard deviation of the results obtained by testing the mini-crane environment with 10 different random seeds over 25 episodes, after identifying the best hyperparameters. The reported metrics in the table do not reflect per-episode scores; instead, they summarize the success rate (*goal reached*) and the number of *collisions* with the ground or walls. Subsequently, the base distribution was replaced and the model retrained using the same hyperparameters. The table shows, on the left, the new results after swapping the distributions.

Dist	Rank	Target reached μ±σ	Collisions μ±σ	AUC μ±σ	Swap dist	Target reached μ±σ	Collisions μ±σ
sq. Gauss	#1	0.94±0.05	0.0±0.0	181.24±38.02	Kumaras	0.97±0.04	0.01±0.02
sq. Gauss	#2	0.93±0.05	0.02±0.04	114.26±14.77	Kumaras	0.93±0.05	0.01±0.02
sq. Gauss	#3	0.99±0.016	0.0±0.0	141.85±35.06	Kumaras	0.88±0.04	0.01±0.02
Kumaras	#1	0.84±0.10	0.004±0.01	93.16±3.60	sq. Gauss	0.57±0.17	0.03±0.05
Kumaras	#2	0.82±0.14	0.02±0.03	92.33±2.43	sq. Gauss	0.63±0.17	0.02±0.03
Kumaras	#3	0.80±0.13	0.04±0.04	93.72±1.65	sq. Gauss	0.65±0.13	0.04±0.07
Beta	#1	0.78±0.14	0.024±0.03	94.70±2.10	no swap	—	—
Beta	#2	0.87±0.07	0.004±0.01	95.75±1.68	no swap	—	—
Beta	#3	0.79±0.11	0.016±0.03	89.07±6.33	no swap	—	—

Swapping hyperparameters from the Kumaraswamy to the squashed Gaussian distribution leads to a performance drop, while swapping the distribution from the squashed Gaussian to the Kumaraswamy leads to very similar performance, improving target-reaching rates but increasing collision frequency marginally.

These results suggest that both distributions can be effectively tuned to achieve competent and reliable behavior in the mini-crane environment. While the squashed Gaussian shows slightly higher success rates and marginally fewer collisions in some configurations, the agent based on Kumaraswamy also delivers strong performance, achieving high target-reaching rates with low collision frequencies. On the other hand, the performance delivered by the Beta and Kumaraswamy distributions is very similar.

One of the factors contributing to the observed performance decline when transitioning from the Kumaraswamy distribution to the squashed Gaussian distribution is the tendency of the TPE algorithm to identify networks with easy architecture when optimizing a squashed Gaussian agent. The top-ranking results for the squashed Gaussian demonstrate that the network capacity for the actor and critic is minimal, with each comprising two dense layers and 64 units.

Conversely, the same TPE algorithms demonstrate a propensity to favor more intricate network architectures for Kumaraswamy-based agents, characterized by two dense layers with 128 units for the actor and 256 or 512 units for the critic. It appears that the utilization of a higher capacity neural network engenders a rational decline in the performance of the squashed Gaussian agent.

In the context of the LunarLander environment, it has been observed that there is a negligible disparity in performance metrics when transitioning from the squashed Gaussian to the Kumaraswamy and *vice versa*. A thorough analysis of the optimal hyperparameters for both configurations revealed a striking similarity in the neural network architecture across both environments. The TPE algorithm identified a best configuration that is particularly straightforward in both cases. The actor network consists of two dense layers, each containing 64 units, while the critic network consists of two layers, with 128 units in each layer.

Even though the squashed Gaussian is more commonly used and easier to implement in standard Reinforcement Learning frameworks, the Kumaraswamy distribution is a competitive alternative. The swap test from one distribution to another while retaining optimized hyperparameters maintains a high level of performance, underlying the robustness of both policy types.

This observation supports greater flexibility in policy design for Reinforcement Learning agents: while implementation simplicity may favor squashed Gaussian distributions, Kumaraswamy-based models offer comparable effectiveness and can serve as an equally viable option for continuous control tasks such as mini-crane controlling application. For this reason, the Kumaraswamy distribution should be considered when exploring novel architectures or addressing specific application requirements.

Interestingly, an inverted variability pattern can be seen in the training and evaluation stages in the Tables 5, 6; Figure 7. During training, the Kumaraswamy policy produced smoother learning curves (i.e., smaller across-seed standard deviation) than the squashed Gaussian, suggesting more consistent update dynamics. However, during testing, Kumaraswamy’s policy exhibited greater variability in episode returns between seeds, while squashed Gaussian exhibited lower test-time variance. These two measures likely reflect different sources of randomness. Training-time variance quantifies differences in update trajectories (i.e., gradient/update stability), while test-time variance is dominated by sampling from the learned stochastic policy and sensitivity to initial conditions. The Kumaraswamy parameterization natively models bounded and potentially concentrated action distributions. This may yield steadier updates, but it also produces higher per-episode sampling variability (i.e., sharper modes) at evaluation time. Conversely, the squashed Gaussian produces smoother sampling noise at test time despite slightly less stable updates during training. Future work will address this causal analysis in more detail (gradient-variance diagnostics, LR ablations, and per-action histograms).

As illustrated in Figure 7, the training curves of both the Kumaraswamy and Beta distributions appear to be smoother than those of the squashed Gaussian. This phenomenon can be attributed to the gradient saturation induced by the tanh squash technique employed by the squashed Gaussian. The Kumaraswamy and Beta distributions are both inherently constrained within the interval [0,1] and require only a linear function to map the action space from [0,1] to [−1,1] . A potential explanation for this phenomenon could be the differentiable inverse cumulative distribution function (CDF) of the Kumaraswamy distribution, as expressed by Equation 3 or Equation 4. This results in more stable logarithmic probability gradient updates, which in turn lead to smoother policy updates.

**FIGURE 8:**
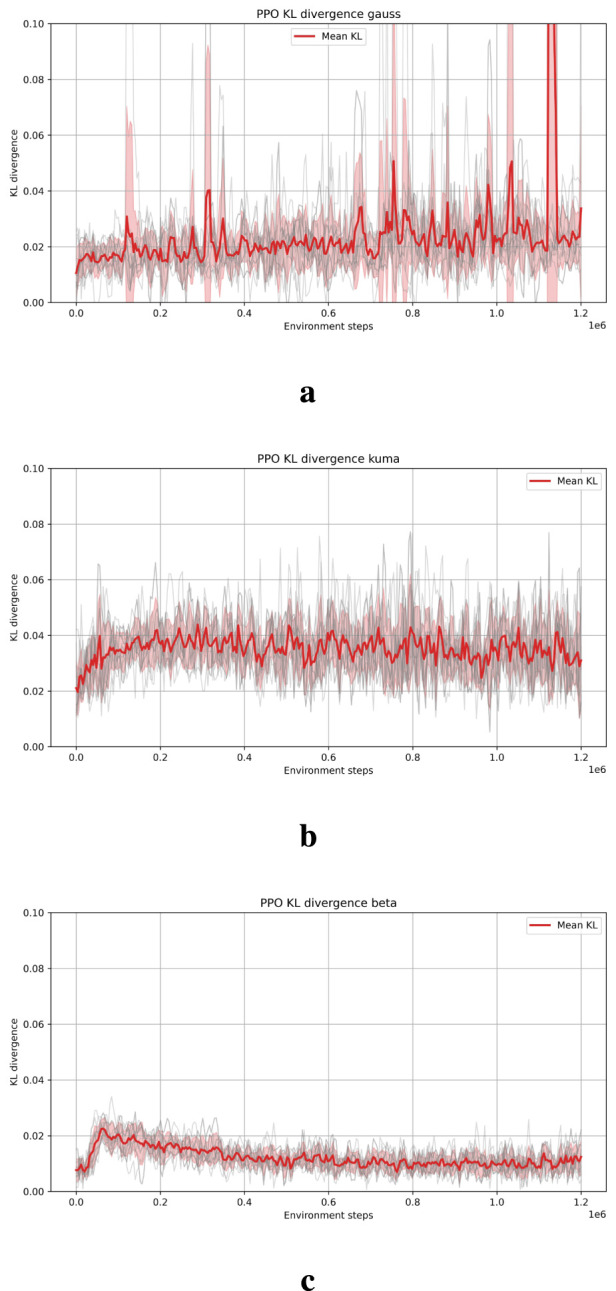
KL divergence of the three distributions for the mini-crane environment. **(a)** Squashed Gaussian distribution. **(b)** Kumaraswamy distribution. **(c)** Beta distribution.

In order to understand this behavior, the KL divergence between consecutive policies has been monitored and used as an indicator of update stability. As illustrated in the following Figure 8, where the Kullback-Leibler divergence for the best hyperparameters across 10 random seeds for the mini-crane environment have been collected, the Beta distribution produces smoother and more stable KL trajectories compared to the squashed Gaussian, indicating a greater degree of controlled policy updates. On the other hand, the Kumaraswamy KL divergence appears slightly less noisy than the squashed Gaussian. This finding provides a potential explanation for the observation of smoother training curves and improved learning stability of the agent.

**FIGURE 9:**
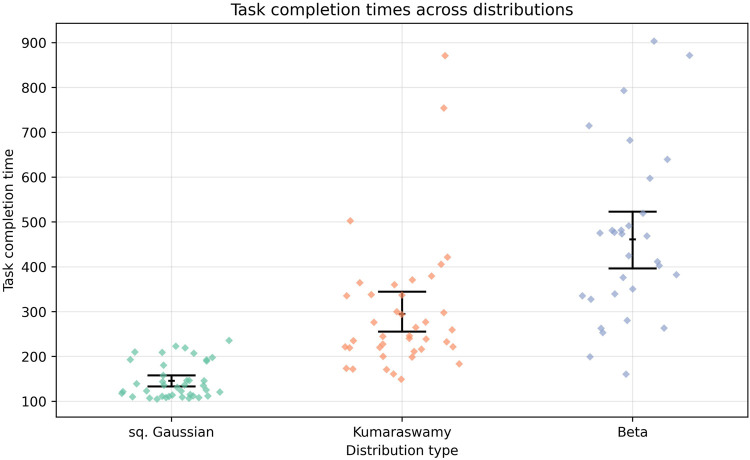
Episode completion time in the mini-crane environment for all three distribution types. Agents must reach the target within a maximum of 2048 steps per episode.

An important observation relates to the time that each agent requires to complete an episode. A comparison of episode durations indicates that agents that utilize the Kumaraswamy distribution require more time than those employing a squashed Gaussian policy, as shown in Figure 9. Conversely, agents based on the Beta distribution demonstrate the longest episode durations. This phenomenon can be attributed to the elevated computational complexity inherent in the calculation of logarithmic and entropy functions for the Beta distribution, as previously discussed in the introduction and reported in Table 1. It is imperative to note that each episode is limited by a maximum of 2,048 steps. Furthermore, the increase in steps required by all three distributions exhibits an almost linear trend.

It should be noted that an agent based on the Kumaraswamy distribution, when trained using optimized hyperparameters for a squashed Gaussian, achieved a success rate nearly identical (97%) to that reported by Andersson et al. (2021) for their log manipulator task. However, Andersson et al. relied on Curriculum Learning over roughly 20 million training steps to reach this level of performance. In contrast, the approach proposed in this study achieved similar results after only 1.2 million timesteps.

Agents employing Beta distributions exhibited significantly higher computational demands, requiring four-to-five times longer execution than those using squashed Gaussians and about twice as long as those with Kumaraswamy policies, which has important implications when considering deployment on embedded platforms with limited hardware resources.

Furthermore, both Kumaraswamy and Beta-based policies tend to produce smaller incremental changes during policy updates compared with squashed Gaussians, a characteristic potentially beneficial in real-world robotics, where frequent or abrupt control actions can cause increased wear or mechanical stress on hardware components. Future research should further investigate how different policy distributions influence action smoothness and long-term system reliability during deployment in physical environments.

## 6 Conclusion

This study has demonstrated the feasibility and benefits of employing the Kumaraswamy distribution as a policy parameterization in on-policy reinforcement learning algorithms for continuous control tasks within construction robotics. By training an agent to operate a simulated mini-crane using PPO with Kumaraswamy-based stochastic policies, this study has shown that this approach yields robust performance comparable to established squashed Gaussian and Beta distributions. Notably, the Kumaraswamy distribution offers practical advantages: it enables closed-form log-probability calculations crucial for gradient-based updates, admits efficient entropy approximations, and results in lower computational overhead during training compared to the Beta distribution: an important consideration for real-time or embedded applications. The results in the previous chapters indicate that the Kumaraswamy distribution is not only a theoretically sound alternative but also provides tangible implementation benefits over the Beta distribution. It achieves competitive task success rates while simplifying policy network design and reducing episode completion times (compared to the Beta distribution).

Looking forward, several promising research directions emerge:

- Hyperparameter Search Analysis:: Future work will involve employing alternative hyperparameter optimization methods to investigate why TPE tends to suggest more complex architectures for agents utilizing the Kumaraswamy distribution.
- Action Magnitude Analysis: Systematically analyzing how different policy distributions affect action magnitudes will be crucial, as this directly impacts hardware wear and overall system stability in real-world deployments.
- Sim-to-Real Transfer: The next phase will involve deploying the learned framework on an actual mini-crane platform, where direct access to target coordinates may be unavailable. Integrating UWB-based distance measurement into the reward function will facilitate this transition.
- Safe Training and Dynamics Modeling: To mitigate risks associated with abrupt or unsafe actions during early-stage exploration of physical systems, the next study will focus on developing a neural network-based dynamic model of the crane. This model can serve as a surrogate environment for pre-training agents or for simulating mechanical stress constraints.
- Task Complexity and Recurrent Policies: Further studies may also extend task complexity by introducing sequential goals or adopting recurrent architectures (e.g., LSTM/GRU) within PPO to address partial observability, particularly relevant when full-state information (such as joint velocities) is unavailable.

In summary, this work establishes the Kumaraswamy distribution as a viable and efficient alternative for continuous-action reinforcement learning in robotics. Continued research along these lines will deepen our understanding of its properties and support safer and more adaptable deployment of RL agents in complex real-world environments.

## Data Availability

The datasets presented in this article are not readily available because It is a reinforcement learning environment. The data are generated on the go. Requests to access the datasets should be directed to Davide Picchi, picchi@ip.rwth-aachen.de.

## Footnotes

1 The α and β parameters are used in this study, even for the Kumaraswamy distribution, despite the fact, that a and b are used commonly in mathematics

2
https://cyberbotics.com/
