# Supplementary material for: Exploiting the Kumaraswamy distribution in a reinforcement learning context
Source: Front Robot AI. 2025 Oct 30;12:1589025. doi: 10.3389/frobt.2025.1589025 (PMC12611641; doi:10.3389/frobt.2025.1589025)
Supplement: Supplementary file 1 [file Supplementaryfile1.pdf]

Appendix

1 APPENDIX

2 HYPERPARAMETERS

The subsequent tables (Tab. S1 and Tab. S2) summarize the top-3 hyperparameters that were identified using Optuna and the TPE algorithm. The parameter search was executed by executing 100 trials in the following hardware configuration:

- CPU: AMD Ryzen 9 7950X3D, 16-core processor
- GPU: NVIDIA GeForce RTX 4080
- RAM: 64 GB DDR4

The software environment was:

- Operating System: Ubuntu 24.04 LTS
- Python: 3.12.11
- PyTorch: 2.8.0, CUDA: 12.9, cuDNN: 9.1.0
- Gymnasium: > 1.0
- Optuna: 3.2.0

2.1 LunarLander environment

Rank	Hyperparameter	sq. Gaussian	Kumaraswamy	Beta
	N	1024	1024	1024
Top-1	Batch size	256	128	256
	Entropy coefficient c_2	0.0026	0.0035	0.0032
	λ_{gae}	0.994	0.952	0.9598
	Discount factor γ	0.99	0.996	0.998
	Actor layers	[64, 64]	[64, 64]	[64, 64]
	Critic layers	[64, 64]	[64, 64]	[64, 64]
	Actor learning rate	0.000238	0.00027	$8.14e-05$
	Critic learning rate	0.00178	0.00496	0.0017
	Policy clip	0.20	0.20	0.198
	Value function coeff. c_1	0.494	0.497	0.532
	N	1024	1024	1024
Top-2	Batch size	128	128	256
	Entropy coefficient c_2	0.0025	0.0046	0.0032
	λ_{gae}	0.9487	0.9459	0.9590
	Discount factor γ	0.9938	0.9973	0.9976
	Actor layers	[64, 64]	[64, 64]	[64, 64]
	Critic layers	[128, 128]	[128, 128]	[64, 64]
	Actor learning rate	0.00027	$8.902e-5$	$8.957e-5$
	Critic learning rate	0.00157	0.00172	0.0017
	Policy clip	0.1956	0.2	0.1936
	Value function coeff. c_1	0.4835	0.4553	0.5245
	N	1024	1024	1024
Top-3	Batch size	256	128	256
	Entropy coefficient c_2	0.0026	0.0028	0.0033
	λ_{gae}	0.9478	0.9538	0.9596
	Discount factor γ	0.9925	0.9967	0.9989
	Actor layers	[64, 64]	[64, 64]	[64, 64]
	Critic layers	[128, 128]	[128, 128]	[64, 64]
	Actor learning rate	0.00027	0.00022	$8.817e-5$
	Critic learning rate	0.0018	0.00138	0.00174
	Policy clip	0.1970	0.2	0.1949
	Value function coeff. c_1	0.499	0.4544	0.5253

Table S1. Top-3 hyperparameters for the **LunarLander** environment.

2.2 Mini-crane environment

Rank	Hyperparameter	sq. Gaussian	Kumaraswamy	Beta
	N	1024	1024	1024
Top-1	Batch size	128	256	512
	Entropy coefficient c_2	0.00	0.0582	0.024
	λ_{gae}	0.95	0.7034	0.809
	Discount factor γ	0.984	0.987	0.996
	Actor layers	[128, 128]	[128, 128]	[64, 64]
	Critic layers	[128, 128]	[256, 256]	[128, 128]
	Actor learning rate	0.00037	0.00027	0.00013
	Critic learning rate	0.00029	0.00204	0.005
	Policy clip	0.20	0.237	0.1509
	Value function coeff. c_1	0.50	0.6328	0.2308
	N	1024	1024	1024
Top-2	Batch size	128	512	512
	Entropy coefficient c_2	0.00	0.0879	0.0237
	λ_{gae}	0.95	0.7478	0.8179
	Discount factor γ	0.9899	0.9906	0.9945
	Actor layers	[128, 128]	[256, 256]	[64, 64]
	Critic layers	[128, 128]	[512, 512]	[128, 128]
	Actor learning rate	0.00047	0.00058	0.00024
	Critic learning rate	0.00018	0.00122	0.00478
	Policy clip	0.2001	0.2209	0.1524
	Value function coeff. c_1	0.502	0.7992	0.6578
	N	1024	1024	1024
Top-3	Batch size	128	512	512
	Entropy coefficient c_2	0.0001	0.051	0.0259
	λ_{gae}	0.9501	0.7255	0.8198
	Discount factor γ	0.9822	0.9864	0.9989
	Actor layers	[128, 128]	[128, 128]	[64, 64]
	Critic layers	[128, 128]	[128, 128]	[128, 128]
	Actor learning rate	0.00037	0.00099	0.00122
	Critic learning rate	0.00016	0.00114	0.0049
	Policy clip	0.1999	0.2366	0.1532
	Value function coeff. c_1	0.5009	0.6617	0.2082

Table S2. Top-3 hyperparameters for the **mini-crane** environment.

2.3 Environment parameters

Coefficient	Description	Value
c_3	Scaling factor for the variation of the Euclidean distance between the hook and the goal position	10.0
c_4	Bonus when goal is reached	50.0
c_5	Penalty when collision occurs	-10.0

Table S3. Reward function coefficients used for the mini-crane environment.
